# Phobia-specific patterns of cognitive emotion regulation strategies

**DOI:** 10.1038/s41598-023-33395-6

**Published:** 2023-04-13

**Authors:** Andras N. Zsido, Andras Lang, Beatrix Labadi, Anita Deak

**Affiliations:** 1 Institute of Psychology, University of Pécs, 6 Ifjusag Street, Pécs, Baranya 7624 Hungary; 2 Szentágothai Research Centre, University of Pécs, Pécs, Hungary

**Keywords:** Psychology, Evolutionary theory, Health care

## Abstract

Although fear plays a vital role in survival, an overly active threat detection system could be maladaptive due to its negative health consequences. Putatively maladaptive emotion regulation (ER) strategies are a core problem in phobias. In contrast, adaptive ER strategies could help downregulate the emotion elicited by a threatening stimulus and decrease anxiety. Yet, the number of studies directly examining the pattern of ER strategies linked to various phobias is still scarce. Thus, this study sought to map the patterns of adaptive and maladaptive ER strategies linked to the three most common phobias (social, animal, and blood-injection-injury [BII]). A total of 856 healthy participants filled out our survey including self-reported measures of social anxiety, snake-, spider-, BII phobia, and cognitive ER strategies. Structural equation modeling was used to test the effects between the variables. The results show that social anxiety and animal phobia were linked to both adaptive and maladaptive ER strategies, while BII was only associated with maladaptive ones. Further analyses showed that the most prominent ER strategies differed by subtype. This is in line with previous neuroimaging studies claiming that the neurocognitive mechanisms underlying phobias are also different. Theoretical as well as practical implications are discussed.

## Introduction

Emotions play a vital role in the survival of the human species. To better explain the relation, LeDoux proposed a theory of survival circuits, neural networks that emerged during the evolution to increase the chances of reproduction and survival of the organism^1,2^. One of these, the defensive survival circuit, has been the subject of increasing interest due to its importance in the acquisition and maintenance of various fears and phobias. The defensive circuit is responsible for detecting threats, initiating defensive behaviors (e.g., flight or fight), and supporting physiological adjustments (e.g., increased sympathetic nervous system activity). This automatic detection and response system is paralleled by a second system that generates conscious feelings (i.e., fear), as emphasized by the two-system framework^3^. While threat detection and defensive behaviors are crucial for survival^4,5^, hyperactivation of the defensive systems can cause distress and impairment. For instance, objects that are visually similar to phobia-related objects cause more false alarms (e.g., when someone is startled by something that looks like a snake, but it is just a log). Although it is better to err on the side of caution^6^, the long-term consequences of too many false alarms are heightened sensitivity, enhanced fear, and the maintenance of phobia. Further, automatic emotional reactions, such as fear and avoidance, could persist even in the absence of conscious memory of the original events^7^. However, Individuals can influence the emotions they have and how they experience them by using cognitive emotion regulation (ER) strategies^8,9^. That is, ER is important for keeping fear and other negative emotions at an adaptive level.

The strategies can be subdivided into putatively *adaptive* (acceptance, refocus on planning, positive refocusing, positive reappraisal, putting into perspective) and putatively *maladaptive* (self-blame, blaming others, rumination, and catastrophizing) ones based on whether using the strategy decreases or increases negative feelings, anxiety, and depression^10–12^. A previous study^11^ identified nine cognitive ER strategies individuals use after encountering negative life events (see Supplementary Table 1 for short definitions). ER strategies that are usually associated with adaptive outcomes (such as reductions in the experience of negative affect, increased pain tolerance, effective interpersonal functioning, and diminished maladaptive cardiac reactivity) are termed adaptive ER strategies. In contrast, ER strategies that are generally associated with maladaptive outcomes (e.g., rebounds in negative affect, memory difficulties, increases in sympathetic activation, and diminished autonomic flexibility) are termed maladaptive ER strategies^9^. While strategies falling in the latter category are termed “regulation” strategies, they often result in emotional dysregulation. ER strategies can be maladaptive if they are an unsuccessful attempt to down- or up-regulate emotions, down-regulate emotions in the short term but maintain or increases them in the long term, or is an attempt to up-regulate emotions when it would be more maladaptive to down-regulate them.

ER strategies, gaining an increased interest in the past decades, are also an integral part of the biases of the cognitive system toward threats. Both adaptive and maladaptive ER strategies could predict the severity of fears, phobias, and existing symptoms^10,13,14^. On the one hand, dysregulation (such as catastrophizing) of emotions, such as fear and disgust, has been shown to be a core problem in various phobias^15–17^. On the other hand, targeting ER skills in treatment, such as decreasing the use of maladaptive ER strategies while teaching adaptive ones in treatment, could increase the success rate of the therapy^8,15,18^. For instance, the cognitive change in the meaning of an emotion-eliciting situation (reappraisal) has been shown to reduce negative feelings^19^. Understanding the adaptive and maladaptive cognitive regulatory processes associated with various phobia subtypes may help clinicians identify treatment targets for them^20^.

A recent study^21^ argues that the pattern of ER strategies involved in phobias is different from subtype to subtype, possibly because the neurocognitive mechanisms underlying them are also different^22,23^. These studies generally compared the brain activity correlates of stimulus exposure between patient groups. For instance, in a study^22^ where participants with snake (animal subtype) or dental (BII subtype) phobia saw phobia-specific videos in an MR scanner, the results showed a different activation pattern for the two subtypes. The authors argue that snake phobia is associated with limbic and paralimbic structures, while dental phobias are mainly associated with pre- and orbitofrontal areas. Further, spider phobia was linked to the dorsal anterior cingulate and anterior insula, while blood-injury-injection (BII) phobia was associated with the thalamus and visual/attention areas such as the occipito-temporo-parietal cortex^24,25^. In contrast, in social anxiety disorder (SAD) the amygdala, insula, hippocampus, and medial prefrontal cortex were identified as core brain areas involved in the development and maintenance of fear^26^. In fact, Caseras and colleagues^25^ argue that although the immediate reaction to phobia-related stimuli is highly similar in the various subtypes of phobia, their results regarding the subtype-specific difference in the dynamics of BOLD responses of the left and right amygdala could point to a difference in emotion regulation processes. In the present investigation, similarly to previous studies^27,28^, we limited the focus of our search to the three most common phobias: animal phobia, BII phobia, and social anxiety disorder with a lifetime prevalence of 3.8%, 3%, and 13%, respectively^29,30^. In various animal phobias, in particular, regard to snake and spider fears, cognitive reappraisal has been identified^31,32^ as a good strategy to regulate negative emotions. In contrast, social anxiety has been linked to all four maladaptive ER strategies, as well as positive reappraisal^33–35^. Further, BII phobia has often been associated with emotion regulation difficulties in brain imaging studies^27,36^ and a general emotion regulation deficit in this disorder^28^. However, to date, to our knowledge, no previous study sought to systematically test the common and specific putatively maladaptive strategies behind emotion dysregulation and putatively adaptive strategies that could be targeted in interventions in the different subtypes of phobias.

In the present study, we sought to show the common relationships between ER strategies and various fears (connected to phobias) and the possible specificity of ER strategies with different fears. Our goal was to use a general sample of healthy individuals assessing them using self-report measurements instead of conducting diagnostic interviews. This allowed us to have a large sample size and to use fears as continuous variables. We did not want to limit the findings to phobic vs. non-phobic individuals but rather include the whole spectrum of fears. Similar to a previous study^27^, we selected the three phobias with the highest lifetime prevalence rates, i.e., animal (indicated by measures of snake and spider phobia), BII, and social anxiety disorder. First, we tested if both adaptive and maladaptive ER strategies play an important role in fears and phobias. We hypothesized that both adaptive and maladaptive ER strategies would be associated with all three subtypes. Thus, we tested a model where adaptive and maladaptive ER strategies predicted measures of animal-, BII phobia, and social anxiety. Second, we also wanted to discover the unique pattern of ER strategies behind various subtypes Further, our research question was whether the ER strategies differed by subtype. To test this, we tested three separate models to identify the pattern of ER strategies linked to the three phobias. Based on previous studies, we expected that catastrophizing and reappraisal would appear as transdiagnostic phenomena.

## Methods

### Participants

We recruited 856 Caucasian participants (739 females), aged 18–80 years (M = 25.96, SD = 10.04) through the Internet by posting invitations on various forums and mailing lists to obtain a non-clinical heterogeneous sample. Table 1 shows the central tendencies of the questionnaires and more details about the sample. None of the respondents reported having been diagnosed with a specific phobia or social anxiety disorder. Subjects participated voluntarily. The required sample size for this experiment was determined by computing estimated statistical power with a conservative approach (RMSEA = 0.05, β > 0.95, alpha = 0.05) using the semPower package for R^37,38^. The analysis indicated a required total sample size of 679; thus, our study was adequately powered. The research was approved by the Hungarian United Ethical Review Committee for Research in Psychology and was carried out in accordance with the Code of Ethics of the World Medical Association (Declaration of Helsinki). Informed and written consent was obtained from all participants.

**Table 1:** Detailed descriptive statistics of the sample including demographic variables and the questionnaires used in the study. *CERQ* Cognitive Emotion Regulation Scale, *SIAS* Social Interaction Anxiety Scale, *SPS* Social Phobia Scale, *SNAQ* Snake Phobia Questionnaire, *SPQ* Spider Phobia Questionnaire, *MFS* Medical Fear Survey.

Variable	Category	Count	
Gender	Females	739	
Residence	Village	174	
	Town	290	
	Large city	116	
	Capital	276	

	Mean	Median	SD
Age	25.96	22	10.04
CERQ self-blame	5.57	5	2.08
CERQ acceptance	6.47	6	2.02
CERQ rumination	6.36	6	2.2
CERQ positive refocusing	5	5	2.17
CERQ refocus on planning	7.09	7	1.88
CERQ positive reappraisal	6.71	7	2.17
CERQ putting into perspective	6.26	6	2.16
CERQ catastrophizing	4.73	4	2.25
CERQ other blame	3.84	4	1.59
SIAS	6.69	5	5.73
SPS	6.24	4	5.86
SNAQ	2.82	1	3.4
SPQ	4.16	3	3.84
MFS injections and blood draws	2.89	1	3.57
MFS sharp objects	1.42	0	2.3
MFS examinations and symptoms	6.34	6	3.69
MFS blood	2.36	1	3.51
MFS mutilation	7.15	7	4.13

### Materials

#### Socio-demographic questions

Socio-demographic questions included age, gender, the size of residence (village, town, large city, capital), and whether they have been diagnosed by a psychiatrist or clinical psychologist as having a specific phobia or social anxiety disorder.

#### Social anxiety

The 6-item version of the Social Phobia Scale (SPS) and Social Interaction Anxiety Scale (SIAS)^39^ measures social phobia. The SPS is dedicated to measuring specific scrutiny fears, while the SIAS measures social interaction-related fears. Items are rated on a 5-point Likert-type scale. The McDonald’s omegas were 0.88 and 0.89.

#### Animal phobia

The 12-item version of the Snake Phobia Questionnaire (SNAQ) and Spider Phobia Questionnaire (SPQ) was used to measure self-report fear and phobia of snakes and spiders^40,41^. SNAQ and SPQ are one-factor scales, that use a dichotomous response format (true; false). ‘True’ responses are summed to yield a score ranging from 0 to 12. Higher scores indicate higher levels of fear of snakes/spiders. In this study, the McDonald’s omega was 0.91 (SNAQ) and 0.92 (SPQ).

#### Blood–injection–injury phobia

The Medical Fear Survey (MFS)^42,43^ measures BII phobia with 25 items assessing medically related fears across five domains: Injections and Blood Draws (IB, 4 items, maximum score = 12), Sharp Objects (SO, 5 items, maximum score = 15), Blood (BL, 5 items, maximum score = 15), Mutilation (MU, 5 items, maximum score = 15), and Examinations and Symptoms (ES, 6 items, maximum score = 18). Participants rate their degree of fear if they were to be exposed to medically related situations described by the items, using a 4-point Likert-type scale, ranging from 0 = No fear to 3 = Intense fear. The McDonald’s omegas were 0.92 (IB) and 0.84 (SO), 0.80 (ES), 0.90 (BL), and 0.83 (MU).

#### Cognitive emotion regulation questionnaire (CERQ)

The 18-item version of the CERQ^44^ measures cognitive strategies that characterize the individual’s style of responding to stressful events. The questionnaire has 9 subscales in total, four subscales measure maladaptive strategies (Self-blame, Rumination, Catastrophizing, and Other blame), and five measure adaptive strategies (Acceptance, Positive refocusing, Refocus on planning, Positive reappraisal, and Putting into perspective). The McDonald’s omegas were 0.73 (Self-blame), 0.79 (Acceptance), 0.83 (Rumination), 0.82 (Positive refocusing), 0.63 (Refocus on planning), 0.75 (Positive reappraisal), 0.77 (Putting into perspective), 0.87 (Catastrophizing), and 0.76 (Other blame).

### Statistical analysis

See Table 1 for descriptive statistics of the sample on all measures used and Supplementary Table 2 for correlational coefficients across all included variables. There were no missing data because the response for all survey items was made mandatory during data acquisition. The survey only saved responses that were fully completed, we have no data on partial completions.

First, to get a more general picture of the relationship between different ER strategies and phobia subtypes we used a pathway model approach within Structural Equation Modelling (SEM). This model can answer (1) how various phobia subtypes are defined by adaptive and maladaptive ER strategies in general and (2) how strongly adaptive and maladaptive ER strategies influence the level of fear. Adaptive and maladaptive ER strategies were entered as predictors and social anxiety, BII, and animal phobia as outcome variables into SEM. We have decided to use SEM because it offers the possibility to use latent variables, and test a hierarchical model of predictors and individual effects of the entered variables. ER strategies and the three phobias were entered as latent variables; measured variables were the total and subscale scores of the relevant questionnaires. We allowed covariation between the three phobia types and also between adaptive and maladaptive ER strategies.

Then, we used three Multiple Indicators Multiple Causes (MIMIC) models to separately test the ER strategy patterns for the three measured phobias. This approach is capable of showing us the phobia-specific pattern of ER strategies, i.e., the exact adaptive and maladaptive ER strategies (out of the nine we measured) that are the most impactful on phobia and play the most important roles in the level of fear. We used the MIMIC model because we were interested in identifying factors that are measured by multiple indicators and examining predictors that cause these factors. Here, ER strategies were used as predictors (entered as measured variables), and the outcome latent variable was a phobia. We performed the SEM analyses using the JASP statistical software version 0.16 for Windows^45^ utilizing the lavaan (v. 0.6-1) package for R^46^ to assess fit measures for our proposed models. We used the diagonally weighted least squares (DWLS) estimator^47^. To evaluate model fit, we used the Chi-square, the comparative fit index (CFI), Bollen’s Incremental Fit Index (IFI), and the root mean square error of approximation (RMSEA) The cutoffs for good model fit were nonsignificant Chi-square^48^, CFI and TLI values of 0.95 or greater^49^, RMSEA value of 0.08 or lower^50^.

### Ethical approval

Ethics approval was obtained from the Hungarian United Ethical Review Committee for Research in Psychology.

### Informed consent

Informed consent was obtained from all individual participants included in the study.

## Results

**Figure 1:**
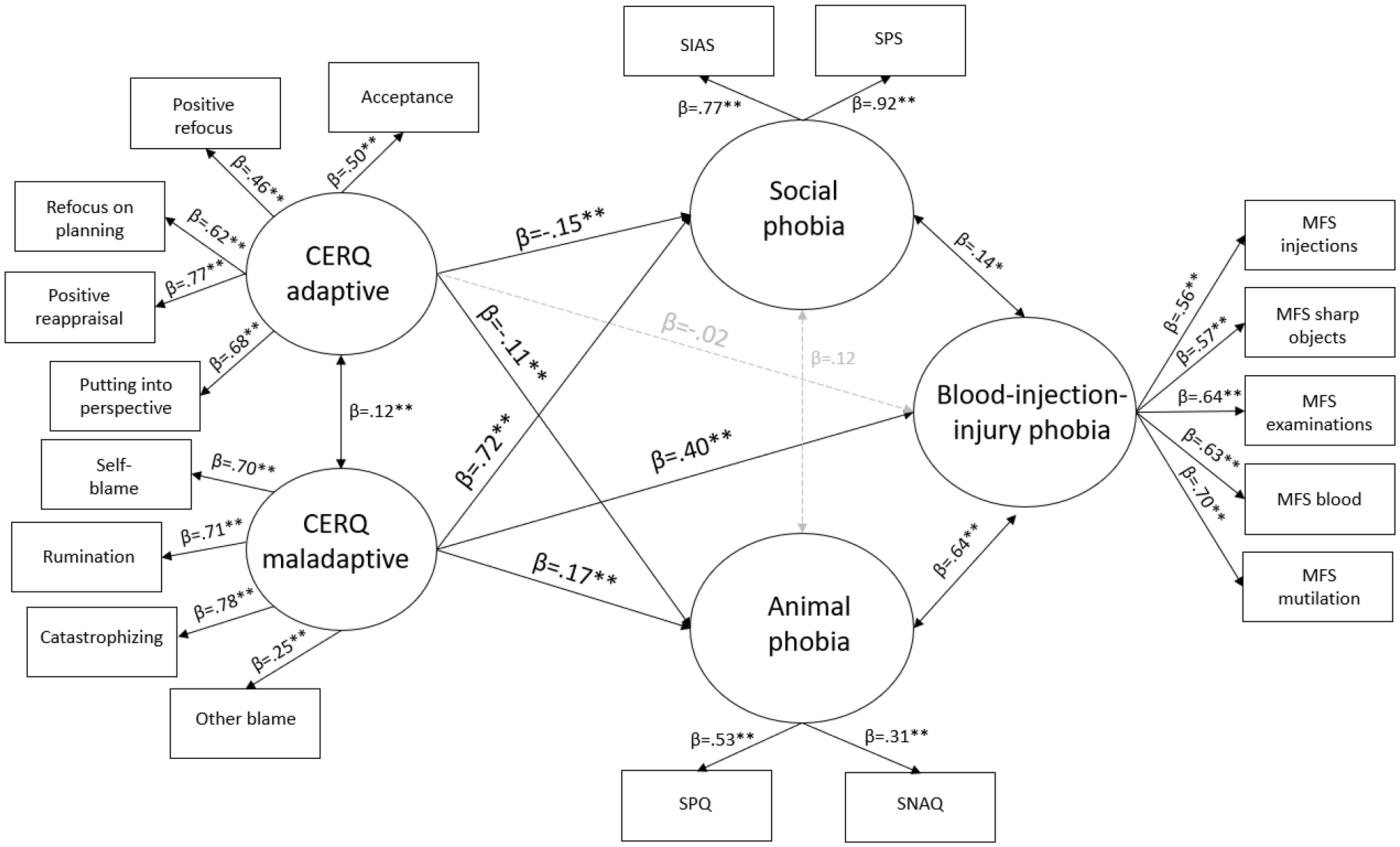
Our proposed model on the connection and background mechanisms of different phobias. All paths included in the analysis are displayed. All reported estimates are the maximum likelihood standardized point estimates. Statistically significant unstandardized point estimates are highlighted in black (*p < 0.05; **p < 0.001). Grey lines indicate a nonsignificant pathway. *CERQ* Cognitive Emotion Regulation Questionnaire, *SPS* Social Phobia Scale, *SIAS* Social Interaction Anxiety Scale, *SNAQ* Snake Phobia Questionnaire, *SPQ* Spider Phobia Questionnaire, *MFS* Medical Fear Survey.

Regarding the pathway model that included all phobias, the test yielded a good model fit (Χ^2^(123) = 381.668, p < 0.001, CFI = 0.953, IFI = 0.953, RMSEA = 0.050, 90% CI = [0.044–0.055], SRMR = 0.055). Animal phobia (R^2^ = 0.16) was predicted by both adaptive (β = − 0.11, p < 0.001, CI: − 0.17 to − 0.05) and maladaptive (β = 0.17, p < 0.001, CI: 0.12 to 0.22) ER strategies. BII phobia (R^2^ = 0.26) was predicted by maladaptive (β = 0.40, p < 0.001, CI: 0.34 to 0.46) but not by adaptive (β = 0.02, p = 0.313, CI: − 0.02 to 0.07) ER strategies. Social anxiety (R^2^ = 0.42) was predicted by both adaptive (β = − 0.15, p < 0.001, CI: − 0.24 to − 0.07) and maladaptive (β = 0.72, p < 0.001, CI: 0.62 to 0.82) ER strategies. See Fig. 1 for the model.

**Figure 2:**
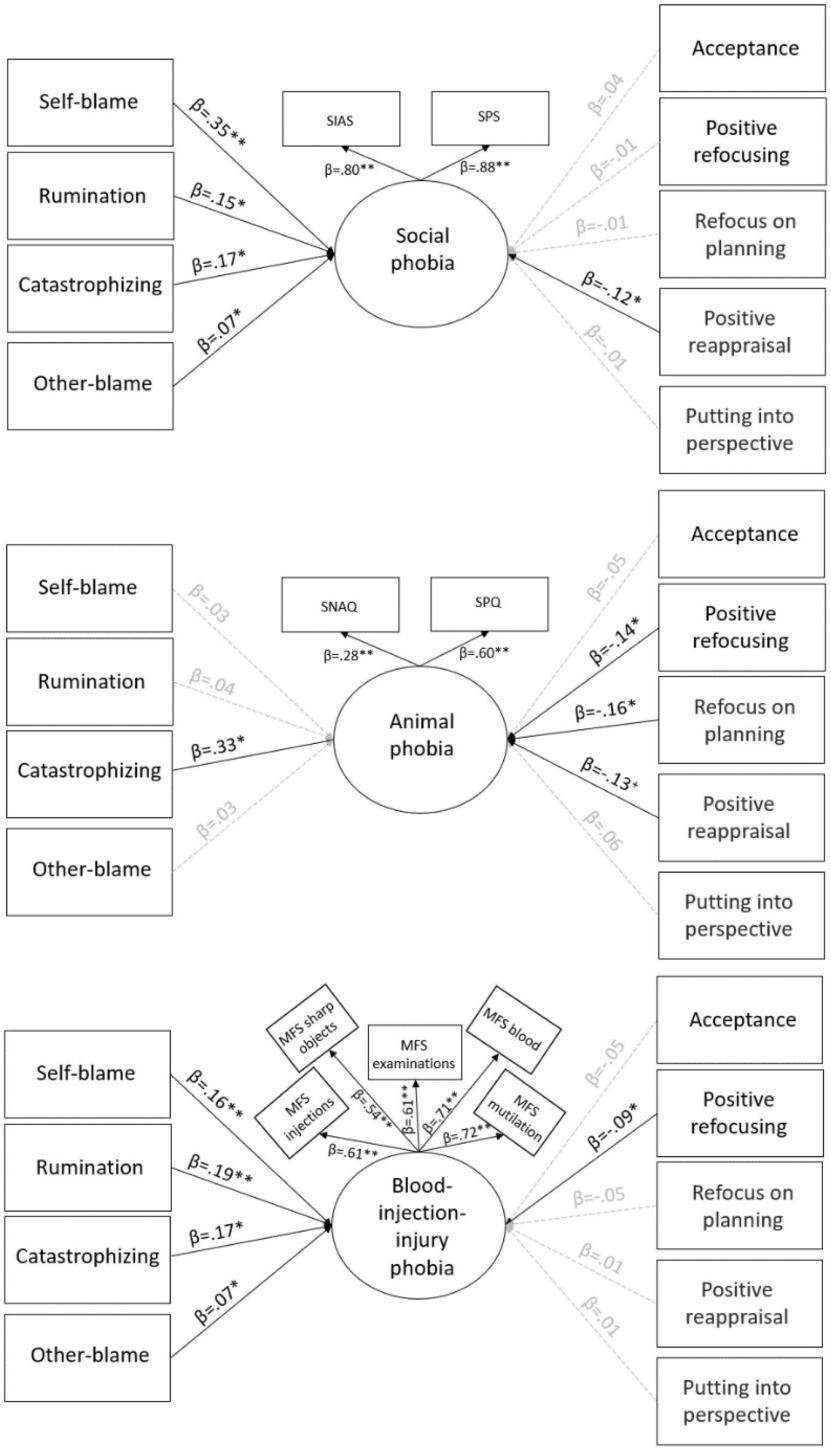
The pattern of emotion regulation strategies behind various phobias. All paths included in the analysis are displayed. All reported estimates are the maximum likelihood standardized point estimates. Statistically significant unstandardized point estimates are highlighted in black (^+^p = 0.05; *p < 0.05; **p < 0.001). Grey lines indicate a nonsignificant pathway. *SPS* Social Phobia Scale, *SIAS* Social Interaction Anxiety Scale, *SNAQ* Snake Phobia Questionnaire, *SPQ* Spider Phobia Questionnaire, *MFS* Medical Fear Survey.

Now we present the results concerning the three MIMIC models, all the tests yielded good model fit. In the first one, we found that *animal phobia* (Χ^2^(8) = 4.539, p = 0.806, CFI = 0.999, IFI = 0.999, RMSEA < 0.001, 90% CI = [0.000–0.025], SRMR = 0.009) was positively predicted by catastrophizing (β = 0.31, p = 0.022), while it was negatively predicted by positive refocusing (β = − 0.14, p = 0.041), refocus on planning (β = − 0.16, p = 0.037), and positive reappraisal (β = − 0.13, p = 0.05). The other effects were nonsignificant. In the second model we found that *BII phobia* (Χ^2^(41) = 78.906, p < 0.001, CFI = 0.979, IFI = 0.979, RMSEA = 0.033, 90% CI = [0.022–0.044], SRMR = 0.035) was positively predicted by self-blame (β = 0.16, p < 0.001), rumination (β = 0.19, p < 0.001), catastrophizing (β = 0.17, p = 0.003), and other-blame (β = 0.07, p = 0.026). It was negatively predicted by positive refocusing (β = − 0.09, p = 0.002). The other effects were nonsignificant. In the third model, we found that *social anxiety* (Χ^2^(8) = 4.445, p = 0.903, CFI = 0.999, IFI = 0.999, RMSEA < 0.001, 90% CI = [0.000–0.017], SRMR = 0.007) was positively predicted by self-blame (β = 0.35, p < 0.001), rumination (β = 0.15, p = 0.004), catastrophizing (β = 0.17, p = 0.007), and other-blame (β = 0.07, p = 0.031). Further, it was negatively predicted by positive reappraisal (β = − 0.12, p = 0.035). The other effects were nonsignificant. Figure 2 shows all three models.

## Discussion

A range of emotions naturally arises when we detect a stimulus that might be threatening. Phobias are associations between negative emotions and objects acquired through conditioning^51^. The dysregulation of emotions is a core problem in various phobias^15–17^. Decreasing the use of putatively maladaptive ER strategies, while teaching putatively adaptive ones in treatment could lessen anxiety, overreaction, and symptoms^8,15,18^. Therefore, our first goal in the present paper was to show that both adaptive and maladaptive cognitive ER strategies are associated with fears (connecting to phobias). Overall, our results suggest that the use of adaptive ER strategies was negatively associated with the severity of animal fears and social anxiety. Further, using maladaptive ER strategies more often was linked to a heightened level of animal-, BII-related fears, and social anxiety^10,13,14^. However, a recent study^21^ showed that the pattern of ER strategies used differs by the subtype of phobia, possibly because of a difference in the neurocognitive mechanisms underlying the subtypes^22–25^. Thus, our second aim was to discover the pattern of the strategies involved in the three subtypes of fears (animal-, BII phobia, and social anxiety) with the highest lifetime prevalence. One of the strengths of our study is that instead of focusing only on one phobia subtype, we assessed the three most common ones. Our results are in line with previous studies^27,28,31–36^ examining different subtypes showing that the pattern of associated ER strategies differs by phobia subtype. These results might have direct implications for clinical practice insofar as treatments should focus on (1) increasing adaptive ERs that are most useful in decreasing fear in that particular subtype (reappraisal in social anxiety, refocusing in BII phobia, and reappraisal, refocusing, and planning in animal phobia), and (2) reducing emotion dysregulation, particularly targeting catastrophizing regardless of phobia subtype. Targeting adaptive ERs by focusing on learning to use these in situations where people encounter the object of their fears is a novel implication of our study. This is in line with previous randomized treatment studies showing that both cognitive and exposure therapies can be used to treat phobias, especially when they focus on learning ER strategies^52,53^.

Catastrophizing was the only ER strategy to emerge in connection to all three subtypes of fears, which is in line with previous studies naming it as a transdiagnostic process across numerous psychopathological disorders, for instance, phobia, panic, obsessive–compulsive disorder, and posttraumatic stress disorder^34,54^. Catastrophizing, with regards to fears and phobias, can result in magnifying a perceived threat, overestimating the frequency of encountering the feared object, and making negative interpretations of ambiguous stimuli (see also Coelho et al.^55^). While the stimuli that set off and maintain the negative cycle of catastrophic thinking are different in each subtype of phobia, the mechanism seems to be similar^27,34^. This resonates well with increased sensitivity to a type of stimulus^6^ and the triggering of the defensive circuit^1^.

The other three maladaptive ER strategies (self-blame, rumination, and other blame) were linked to both BII-related fears and social anxiety, but not to animal fears. Previous studies seemingly agree with a general ER deficit in social anxiety and BII fears. Social anxiety has been associated with all four maladaptive strategies^33–35^, all part of the irrational automatic thoughts appearing in patients with social anxiety disorder in social settings^56^. In a study^28^, BII (but not spider phobia) was linked to general emotion dysregulation. This might be caused by a strong link between BII, health anxiety, and hypochondriacal traits^57^; variables that have been linked to all four maladaptive ER strategies^58^. Similarities between the patterns of brain activation when seeing a phobia-related stimulus in BII phobia and social anxiety as well as their dissimilarity to spider phobia have also been shown in a brain imaging study^27^. Although animal phobia shares a similarity with BII as disgust sensitivity seems a key factor in both^28,57^, the pattern of maladaptive ER strategies that are associated with the two subtypes was different. The fact that animals themselves are agents could be behind the results as one cannot blame themselves or others when encountering one. Similarly, ruminating about the causes and consequences of an encounter does not seem to be relevant in this case. Nevertheless, the vast majority of previous studies focused only on emotional dysregulation while adaptive ER strategies, as possible means of prevention and intervention received little attention. The second strength of our study is that we also sought to discover the adaptive ER strategies linked to the different fears connected to phobia subtypes.

Regarding adaptive ER strategies, animal fears were linked to refocusing, planning, and reappraisal, while the other two subtypes were only linked to one strategy (BII phobia to refocusing, social anxiety to reappraisal). Acceptance and putting into perspective were not connected to any of the subtypes. This is in line with the small number of previous results showing the importance of positive reappraisal both in animal phobia^31,32^ and social anxiety^33^. In our study, animal fear was linked to refocusing, planning, and reappraisal. If people encounter the animal of their fear, they can plan on how to handle the situation (e.g., removing a spider from the room), and think of more pleasant things or generate positive interpretations (“at least it did not bite me”). Using the same strategies might be harder for social and BII-related situations because they are accompanied by the feeling of lack of control (i.e., planning)^59,60^. Instead, in BII-related situations one can distract themselves by thinking of other things, a method that has been shown to be useful in reducing BII fears, increasing approach behavior, and feeling in control^61^. While the generation of positive interpretations may be more helpful and feasible in social settings, and can also result in greater liking of that individual by their peers and sharing emotions more frequently^62^. Interestingly, in the SEM only BII fear was not associated with adaptive ER strategies (where they were used as a latent variable), while in the MIMIC model social anxiety and BII fear both had similarly weak associations with adaptive ER strategies (where they were entered individually). The SEM highlights that adaptive ERs have a role in reducing the level of fear and the MIMIC shows which individual strategies have the greatest effect. Therefore, our results could mean that in these cases adaptive ER strategies are associated with positive outcomes mostly if they are used conjointly. While using only one of them may still help reduce fear, learning and practicing the other strategies should not be neglected to overcome excessive fear.

Limitations of the study include that although we used structural equation modeling for statistical analysis, our study was still correlational in design. Future studies should try and gather longitudinal data, and possibly test if targeting the maladaptive and adaptive strategies our models suggest would decrease the level of fear in a clinical setting. Another important aspect could be, in future studies, the question of probable gender differences. The vast majority of our sample consisted mostly of females, therefore the results might not be universally true for both sexes. Further, cognitive ER strategies were measured in this study concerning stressful situations in general. Future studies might profit from using CERQ with instruction specific to stressful encounters with potentially threatening stimuli (i.e., strangers, BII, and potentially dangerous animals) to measure situation-specific ER strategies. Furthermore, in the present study, we only targeted ER strategies. It would be important for future studies to explore whether similar phobia-specific patterns could be found regarding behavioral or interpersonal strategies.

In conclusion, we showed that both adaptive and maladaptive cognitive ER strategies are associated with fears and phobias. Further, we found that the pattern of associated strategies is not the same across subtypes. This is in line with previous studies^22–25^ suggesting that the neurocognitive mechanisms underlying the various phobia subtypes are also different. Our results may also provide a framework for potential—most favorably cognitive-behavioral-based—interventions to avoid the formation of severe psychopathological consequences. Catastrophizing seems to appear as a transdiagnostic process across all phobia subtypes; the first step of clinical treatments could be reducing the use of that strategy. Similar to a previous study^15^, our results might suggest that interventions should focus on teaching adaptive emotion regulation strategies that can be used to prevent the emotion (associated with the object of phobias) to be formed. In the case of phobias, our results suggest that treatments should focus on different adaptive strategies based on the object of phobic fear. To further understand the emotional mechanisms underlying the different phobia subtypes, future research should use longitudinal methods as well as experimental paradigms along with physiological measures.

## Supplementary Information

Supplementary Table 1.

Supplementary Table 2.

## Data Availability

The data that support the findings of this study are available from the corresponding author upon reasonable request.
