# Supplementary material for: Phobia-specific patterns of cognitive emotion regulation strategies
Source: Sci Rep. 2023 Apr 13;13:6105. doi: 10.1038/s41598-023-33395-6 (PMC10102078; doi:10.1038/s41598-023-33395-6)
Supplement: Supplementary file 1 — Supplementary Table 1. [file 41598_2023_33395_MOESM1_ESM.docx]

**Supplementary Table 1** – The brief definitions of the nine cognitive emotion regulation strategies measured in the current study.

|  | **Cognitive emotion regulation strategy** | **Short definition** |
| --- | --- | --- |
| Adaptive |  |  |
|  | Acceptance | Thoughts of resignation and acceptance to what has happened. |
|  | Refocus on Planning | Thoughts about what to do and how to handle the current negative event. |
|  | Positive Refocusing | Thoughts about positive, happy and pleasant experiences instead of thinking about current negative events. |
|  | Positive Reappraisal | Thoughts of giving the current event a positive meaning in terms of personal growth. |
|  | Putting into Perspective | Thoughts that relativize and downgrade the importance current negative event compared to other events. |
|  |  |  |
| Maladaptive |  |  |
|  | Self-blame | Thoughts that put the blame on oneself for what one has experienced. |
|  | Blaming others | Thoughts of putting the blame of on the environment or another person for what one has experienced. |
|  | Rumination | Thoughts about the feelings and thoughts that are associated with the negative event. |
|  | Catastrophizing | Thoughts of explicitly emphasizing the terror and negativity of the experience. |
