# Supplementary material for: Phobia-specific patterns of cognitive emotion regulation strategies
Source: Sci Rep. 2023 Apr 13;13:6105. doi: 10.1038/s41598-023-33395-6 (PMC10102078; doi:10.1038/s41598-023-33395-6)
Supplement: Supplementary file 2 — Supplementary Table 2. [file 41598_2023_33395_MOESM2_ESM.docx]

Supplementary Table 2 – Pearson correlation coefficients between the questionnaires used in the study. CERQ = Cognitive Emotion Regulation Scale; SB = Self-Blame; AC = Acceptance; RU = Rumination; REF = Positive refocusing; RP = Refocus on planning; REA = Positive reappraisal; PP = Putting into perspective; CA = Catastrophizing; OB = Other blame; SIAS = Social Interaction Anxiety Scale; SPS = Social Phobia Scale; SNAQ = Snake Phobia Questionnaire; SPQ = Spider Phobia Questionnaire; MFS = Medical Fear Survey; IB = Injections and Blood Draws; SO = Sharp Objects; BL = Blood; MU = Mutilation; ES = Examinations and Symptoms.

|  | | | | | | | | | | | | | | | | | | | | | | | | | | | | | | | | | | | | | |
| --- | --- | --- | --- | --- | --- | --- | --- | --- | --- | --- | --- | --- | --- | --- | --- | --- | --- | --- | --- | --- | --- | --- | --- | --- | --- | --- | --- | --- | --- | --- | --- | --- | --- | --- | --- | --- | --- |
|  |  |  |  |  |  |  |  |  |  |  |  |  |  |  |  |  |  |  |  |  |  |  |  |  |  |  |  |  |  |  |  |  |  |  |  |  |  |
|  | | **CERQ SB** | | **CERQ AC** | | **CERQ RU** | | **CERQ REF** | | **CERQ RP** | | **CERQ REA** | | **CERQ PP** | | **CERQ CA** | | **CERQ OB** | | **MFS IB** | | **MFS SO** | | **MFS ES** | | **MFS BL** | | **MFS MU** | | **SIAS** | | **SPS** | | **SNAQ** | | **SPQ** | |
| 1 |  | — |  |  |  |  |  |  |  |  |  |  |  |  |  |  |  |  |  |  |  |  |  |  |  |  |  |  |  |  |  |  |  |  |  |  |  |
| 2 |  | 0.16 | *** | — |  |  |  |  |  |  |  |  |  |  |  |  |  |  |  |  |  |  |  |  |  |  |  |  |  |  |  |  |  |  |  |  |  |
| 3 |  | 0.49 | *** | 0.18 | *** | — |  |  |  |  |  |  |  |  |  |  |  |  |  |  |  |  |  |  |  |  |  |  |  |  |  |  |  |  |  |  |  |
| 4 |  | 0.02 |  | 0.27 | *** | 0.07 |  | — |  |  |  |  |  |  |  |  |  |  |  |  |  |  |  |  |  |  |  |  |  |  |  |  |  |  |  |  |  |
| 5 |  | 0.21 | *** | 0.24 | *** | 0.36 | *** | 0.25 | *** | — |  |  |  |  |  |  |  |  |  |  |  |  |  |  |  |  |  |  |  |  |  |  |  |  |  |  |  |
| 6 |  | -0.03 |  | 0.38 | *** | 0.12 | *** | 0.28 | *** | 0.54 | *** | — |  |  |  |  |  |  |  |  |  |  |  |  |  |  |  |  |  |  |  |  |  |  |  |  |  |
| 7 |  | -0.04 |  | 0.34 | *** | 0.04 |  | 0.39 | *** | 0.37 | *** | 0.55 | *** | — |  |  |  |  |  |  |  |  |  |  |  |  |  |  |  |  |  |  |  |  |  |  |  |
| 8 |  | 0.53 | *** | 0.06 |  | 0.58 | *** | 0.01 |  | 0.15 | *** | -0.13 | *** | -0.11 | ** | — |  |  |  |  |  |  |  |  |  |  |  |  |  |  |  |  |  |  |  |  |  |
| 9 |  | 0.03 |  | 0.04 |  | 0.15 | *** | 0.17 | *** | 0.11 | ** | 0.06 |  | 0.10 | ** | 0.28 | *** | — |  |  |  |  |  |  |  |  |  |  |  |  |  |  |  |  |  |  |  |
| 10 |  | 0.19 | *** | 0.03 |  | 0.19 | *** | 0.05 |  | 0.06 |  | -0.01 |  | 0.01 |  | 0.19 | *** | 0.06 |  | — |  |  |  |  |  |  |  |  |  |  |  |  |  |  |  |  |  |
| 11 |  | 0.18 | *** | -0.03 |  | 0.19 | *** | 0.01 |  | 0.02 |  | -0.03 |  | -0.05 |  | 0.22 | *** | 0.08 | * | 0.31 | *** | — |  |  |  |  |  |  |  |  |  |  |  |  |  |  |  |
| 12 |  | 0.28 | *** | 0.05 |  | 0.30 | *** | -0.10 | ** | 0.12 | *** | 0.02 |  | -0.01 |  | 0.31 | *** | 0.15 | *** | 0.32 | *** | 0.30 | *** | — |  |  |  |  |  |  |  |  |  |  |  |  |  |
| 13 |  | 0.18 | *** | 0.04 |  | 0.23 | *** | 0.06 |  | 0.06 |  | 0.02 |  | 0.04 |  | 0.23 | *** | 0.09 | * | 0.68 | *** | 0.40 | *** | 0.35 | *** | — |  |  |  |  |  |  |  |  |  |  |  |
| 14 |  | 0.22 | *** | 0.05 |  | 0.23 | *** | -0.10 | ** | 0.10 | ** | -0.01 |  | 0.04 |  | 0.24 | *** | 0.12 | *** | 0.43 | *** | 0.43 | *** | 0.40 | *** | 0.56 | *** | — |  |  |  |  |  |  |  |  |  |
| 15 |  | 0.43 | *** | 0.08 | * | 0.31 | *** | 0.02 |  | 0.08 | * | -0.10 | ** | -0.05 |  | 0.38 | *** | 0.14 | *** | 0.23 | *** | 0.23 | *** | 0.23 | *** | 0.14 | *** | 0.17 | *** | — |  |  |  |  |  |  |  |
| 16 |  | 0.44 | *** | 0.06 |  | 0.39 | *** | -0.02 |  | 0.08 | * | -0.11 | ** | -0.08 | * | 0.43 | *** | 0.11 | *** | 0.24 | *** | 0.27 | *** | 0.33 | *** | 0.20 | *** | 0.22 | *** | 0.70 | *** | — |  |  |  |  |  |
| 17 |  | 0.02 |  | 0.01 |  | 0.05 |  | 0.06 |  | -0.04 |  | -0.03 |  | -0.01 |  | 0.09 | ** | 0.01 |  | 0.10 | ** | 0.17 | *** | 0.12 | *** | 0.18 | *** | 0.24 | *** | 0.02 |  | 0.06 |  | — |  |  |  |
| 18 |  | 0.13 | *** | -0.04 |  | 0.11 | *** | 0.03 |  | -0.06 |  | -0.13 | *** | -0.04 |  | 0.22 | *** | 0.07 | * | 0.19 | *** | 0.20 | *** | 0.24 | *** | 0.21 | *** | 0.21 | *** | 0.13 | *** | 0.23 | *** | 0.17 | *** | — |  |
| Note. * p < .05, ** p < .01, *** p < .001 | | | | | | | | | | | | | | | | | | | | | | | | | | | | | | | | | | | | | |
|  | | | | | | | | | | | | | | | | | | | | | | | | | | | | | | | | | | | | | |
